# Hydrogen Bond-Regulated Rapid Prototyping and Performance Optimization of Polyvinyl Alcohol–Tannic Acid Hydrogels

**DOI:** 10.3390/gels11080602

**Published:** 2025-08-01

**Authors:** Xiangyu Zou, Jun Huang

**Affiliations:** Key Laboratory of High Efficiency and Clean Mechanical Manufacture of Ministry of Education, School of Mechanical Engineering, Shandong University, Jinan 250061, China

**Keywords:** gelation time, hydrogen bond, polyvinyl alcohol, rapid prototyping, tannic acid

## Abstract

Traditional hydrogel preparation methods typically require multiple steps and certain external stimuli. In this study, rapid and stable gelation of polyvinyl alcohol (PVA)-tannic acid (TA)-based hydrogels was achieved through the regulation of hydrogen bonds. The cross-linking between PVA and TA is triggered by the evaporation of ethanol. Rheological testing and analysis of the liquid-solid transformation process of the hydrogel were performed. The gelation onset time (GOT) could be tuned from 10 s to over 100 s by adjusting the ethanol content and temperature. The addition of polyhydroxyl components (e.g., glycerol) significantly enhances the hydrogel’s water retention capacity (by 858%) and tensile strain rate (by 723%), while concurrently increasing the gelation time. Further studies have shown that the addition of alkaline substances (such as sodium hydroxide) promotes the entanglement of PVA molecular chains, increasing the tensile strength by 23% and the fracture strain by 41.8%. The experimental results indicate that the optimized PVA-TA hydrogels exhibit a high tensile strength (>2 MPa) and excellent tensile properties (~600%). Moreover, the addition of an excess of weakly alkaline substances (such as sodium acetate) reduces the degree of hydrolysis of PVA, enabling the system to form a hydrogel with extrudable characteristics before the ethanol has completely evaporated. This property allows for patterned printing and thus demonstrates the potential of the hydrogel in 3D printing. Overall, this study provides new insights for the application of PVA-TA based hydrogels in the fields of rapid prototyping and strength optimization.

## 1. Introduction

Hydrogels are a type of polymeric material formed through different cross-linking mechanisms, such as physical or chemical interactions, and are capable of retaining a large amount of water without dissolving while maintaining good flexibility [1]. They have been widely applied in various fields, including wearable electronic devices [2,3,4,5], biomedical applications [6,7], 3D printing [2,8,9,10] or lubrication [11]. The cross-linking mechanisms of hydrogels can generally be divided into physical cross-linking and chemical cross-linking. Hydrogels formed through chemical cross-linking exhibit robust connectivity. Physical cross-linking forms hydrogels through interactions, such as hydrogen bonding, van der Waals forces, and electrostatic interactions. This process is typically mild and does not require the use of initiators, thus holding great potential for applications. Among them, hydrogels formed through ionic bonding typically exhibit electrical conductivity and are commonly used as materials for fabricating gel sensors. They can also be applied in human-machine interaction systems [12,13]. Unlike chemical cross-linking, physical cross-linking is usually reversible, dynamic, and often self-healing [14]. The physical cross-linking of hydrogels can be disrupted through temperature or pH-changes, thereby achieving recyclability and reducing the potential environmental impact of this material [15,16,17].

Hydrogen bonding is the interaction between the hydrogen atom (hydrogen bond donor) in a molecular fragment X-H and an electron-rich hydrogen bond acceptor [18]. This bonding plays an indispensable role in biological processes, enabling peptide chains to fold into functional proteins and contributing to the formation of the DNA double helix structure [19]. In biomedical applications, He et al. developed a dextrorotatory (D)-peptide supramolecular hydrogel that folds through intermolecular hydrogen bonds or intramolecular hydrogen bonds, which is used to treat postoperative pancreatic fistula [20]. In addition, hydrogen bonds also have important developments and applications in fields such as catalysts and batteries [21,22]. Shi et al. used asymmetric hydrogen bond design to construct a multi-hydrogen-bond network, producing hydrogels with high elasticity and low hysteresis. The hydrogels combine superb mechanical elasticity and toughness, and excellent ionic conductivity [23]. Liu et al. prepared a temperature/pH-sensitive hydrogel via hydrogen bond rearrangement for treating skin wounds [24].

PVA is renowned for its high mechanical strength and biocompatibility. Since Peppas discovered in 1975 that PVA could be gelled through freeze-thaw cycles, research on PVA hydrogels has gradually matured [25,26]. TA is a natural polyphenolic compound. TA’s pyrogallol groups facilitate multiple hydrogen bond formation. Computational studies reveal 25 hydrogen bond donors and 46 acceptors per TA molecule, explaining its superior cross-linking ability. Additionally, TA exhibits strong conjugation effects and biocompatibility [27]. PVA can crosslink with TA through hydrogen bonds to form hydrogels. The incorporation of various substances such as MXenes, chitosan, GelMA, glycerol, nanoparticles, and metal ions into PVA-TA-based hydrogels often effectively enhances their properties, including electrical conductivity, antibacterial activity, antifreeze capability, and tensile strength [25,28,29,30,31,32]. However, the commonly used freeze-thaw cycling method for hydrogel preparation has the disadvantages of a long production cycle and high complexity, and the gelation time is difficult to control. In this study, a facile gelation strategy involving ethanol regulation was employed to achieve tunable gelation rates ranging from 10 s to over 100 s and to optimize the tensile properties of the hydrogels.

This study tries to solve a long-term problem: how to balance rapid gelation, strong mechanics, and good water retention in PVA-TA hydrogels. We use a method based on ethanol evaporation to explore the link between gelation and mechanical properties. This helps us find a suitable way to process the hydrogels for uses like 3D printing and rapid prototyping. By allowing the ethanol within the system to evaporate, the hydrogel gradually forms, enabling control over the gelation speed. By adding different regulators, we get a hydrogel that cures rapidly, retains a high amount of water, and exhibits high mechanical strength. Our findings provide valuable insights into the optimization of PVA-TA hydrogels for advanced applications.

## 2. Results and Discussion

PVA and TA are difficult to dissolve without ethanol as a solvent. The role of ethanol is to disrupt the intermolecular hydrogen bonds and regulate their solubility, and eventually the solution becomes light yellow after totally dissolving.

**Figure 1:**
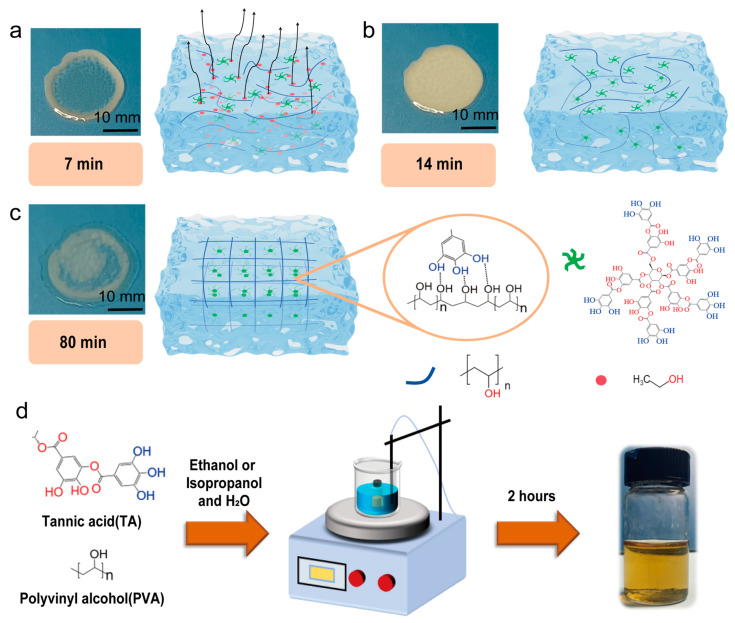
Illustration of the gelation process. (**a**) Ethanol evaporation promotes hydrogel formation. The schematic symbols represent molecules such as ethanol, TA and PVA. (**b**) Disordered network structure post-gelation. (**c**) Ordered network structure upon long-term storage. (**d**) PVA-TA hydrogel sample preparation process.

Figure 1 illustrates the gelation process of the solution. After the solution is applied to the surface of a petri dish, ethanol begins to evaporate, and PVA and TA rapidly form hydrogen bonds. After 7 min, a distinct white precipitate can be observed around the periphery (Figure 1a). This is due to the evaporation of ethanol at the edge of the droplet, which creates a small capillary effect. The subsequent replenishment by the internal liquid leads to a higher deposition of particles, resulting in the coffee ring effect [33]. After 14 min, the ethanol has almost completely evaporated, and the droplet turns entirely white, indicating that PVA and TA have formed a multilayer network structure. However, the internal arrangement is disordered, as shown in Figure 1b, which exhibits low light transmittance. After 80 min, the droplet gradually becomes transparent (Figure 1c), with a concurrent decrease in water content, suggesting a change in the internal cross-linking form. At the initial stage of gelation, the cross-linking of PVA and TA is disordered or low-ordered, resulting in strong light scattering and low light transmittance. Over time, as ethanol continues to evaporate, the cross-linking between PVA and TA becomes more ordered, leading to increased transparency [34]. It is worth noting that when the droplet thickness is relatively large, the ethanol on the surface evaporates first, forming a transparent hydrogel film. The presence of this film makes it difficult for the ethanol inside to evaporate, thereby slowing down the gelation rate. When the hydrogel film is carefully peeled open with tweezers, the ethanol inside will evaporate rapidly (<1 min), resulting in the formation of a milky white hydrogel precipitate. The right side of Figure 1c shows the connection mechanism between PVA and TA after gelation [35]. Figure 1d shows the hydrogel sample preparation process, in which PVA and TA are dissolved in a water bath at 85 °C for two hours.

**Figure 2:**
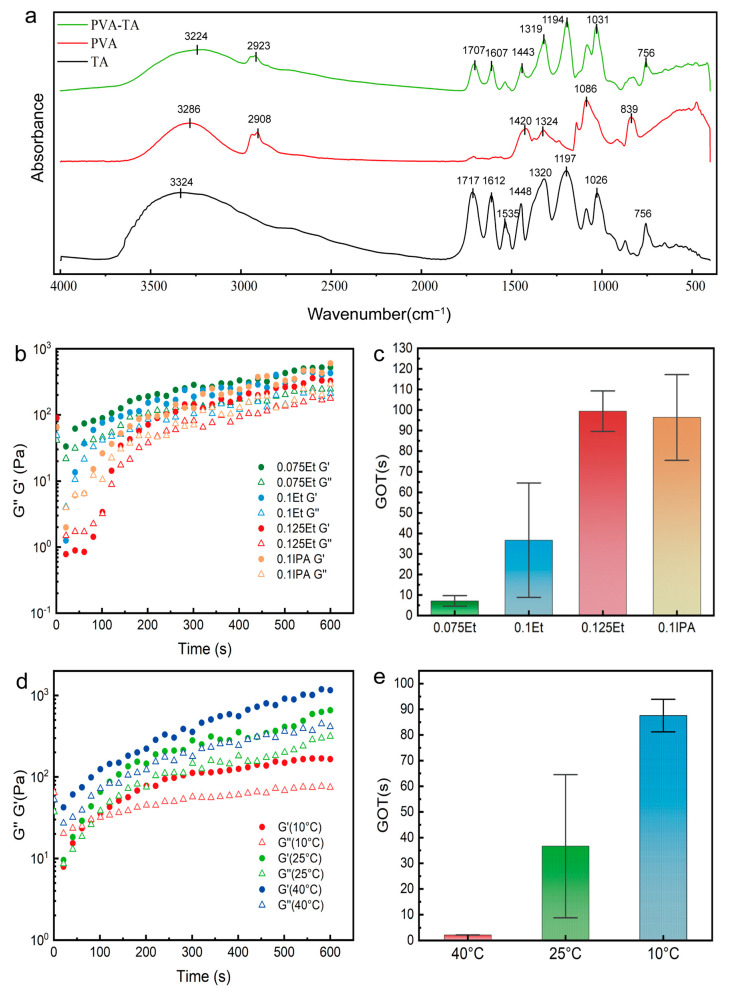
FTIR and rheological testing of PVA-TA hydrogel. (**a**) FTIR spectra of PVA, TA, and PVA-TA hydrogel. (**b**) Changes in G′ and G″ during gelation with different solvents. (**c**) GOT (Gelation Onset Time) with different solvents (n = 3, *p* < 0.05). (**d**) Changes in G′ and G″ during gelation at different temperatures (n = 3~4, *p* < 0.05). The test sample was 0.1Et. (**e**) GOT at different temperatures. The test sample was 0.1Et.

Figure 2a shows the FTIR spectra of PVA, TA, and PVA-TA materials in the wavenumber range of 400–4000 cm^−1^, which are used to verify their chemical compositions. In the spectrum of TA, the absorption peaks at 1717 cm^−1^ and 1612 cm^−1^ correspond to the stretching vibrations of C=O and C=C groups in TA, respectively. The peak at 3324 cm^−1^ is attributed to the stretching vibration of the -OH group. The peaks at 1535 cm^−1^ and 1448 cm^−1^ correspond to the vibrations of the aromatic ring; 1320 cm^−1^ corresponds to the C-O of ester and ether bonds. The absorption peak at 756 may originate from the C-H bond in the aromatic ring. The peaks at 1197 cm^−1^ and 1026 cm^−1^ are assigned to the stretching vibrations of the C-O bonds [36,37,38]. The spectrum of PVA shows a stretching vibration peak of -CH_2_ at 2923 cm^−1^ and a characteristic C-O stretching vibration peak of PVA at 1086 cm^−1^ [16,39]. The absorption peak at 1420 cm^−1^ is the bending vibration of -CH_2_-; the peak at 1324 cm^−1^ is often attributed to the coupled mode of C-H bending vibration and O-H in-plane deformation vibration; 839 cm^−1^ may correspond to structural changes in the crystalline or amorphous regions of PVA.

In the PVA-TA hydrogel, the absorption peaks at 3286 cm^−1^ (PVA) and 3324 cm^−1^ (TA) have shifted to a lower wavenumber at 3224 cm^−1^, indicating the formation of strong hydrogen bonds between PVA and TA [40]. The absorption peak of PVA at 1086 cm^−1^ has shifted to a lower wavenumber and merged with the peak of TA at 1026 cm^−1^ to form a new peak at 1031 cm^−1^. This is due to the formation of intramolecular or intermolecular hydrogen bonds between PVA and TA, resulting in a red shift of the absorption peaks [41]. The absorption peaks at 1707 cm^−1^, 1607 cm^−1^, 1319 cm^−1^, 1194 cm^−1^ and 756 cm^−1^ originate from the absorption peaks of TA, with 1707 cm^−1^ and 1607 cm^−1^ showing slight red shifts, possibly due to C=O participating in hydrogen bond formation. The absorption peak at 1194 cm^−1^ originates from PVA. These findings effectively confirm the composition of the PVA-TA hydrogel. And 1319 cm^−1^ corresponds to the C-O of ester and ether bonds. The infrared spectra indicate that PVA and TA are cross-linked through hydrogen bonds, with the involvement of multiple functional groups, such as -OH and C-O.

The gelation time of PVA and TA was evaluated using rheological testing. Figure 2b shows the changes in storage modulus (G′) and loss modulus (G″) during the gelation process of PVA-TA hydrogels with different solvents. At the beginning of the test, both G′ and G″ are low, and G′ is less than G″, indicating that the hydrogel is more liquid-like at this stage. Taking sample 0.1Et as an example, at 20 s, G′ = 1.28 (Pa); G″ = 4.08 (Pa); at 34 s, G′ = G″ = 6.94 (Pa); when t = 139 s, G′ reaches 101 (Pa), and G″ is 55 (Pa). For sample 0.125Et, G′ and G″ are given at t = 100 (s). As the solvent evaporated from the system, hydrogen bonds continuously formed between PVA and TA, leading to an accelerated increase in the G′. When G′ exceeded G″, the two lines intersected, marking the point at which the solid-like properties of the hydrogel began to dominate over its liquid-like properties. This moment is defined as the gelation onset time (GOT). Figure 2c illustrates the GOT of PVA-TA hydrogels with different solvents. The GOT for the 0.075Et sample averages less than 10 s. As the ethanol content increases, using the 0.1Et sample, the GOT increases significantly to approximately 36.7 s. With the 0.125Et sample, the GOT reaches 99.4 s. This result indicates that the hydrogel only exhibits solid-like properties when the ethanol content in the system is sufficiently low.

Under the condition of maintaining the same amount of substance, replacing ethanol with lower volatility alcohols (e.g., IPA) will increase the GOT of the system. Figure 2c shows that the replacement of 0.1 mol of ethanol by 0.1 mol of isopropanol results in an increase in the GOT by approximately 162.7%. Therefore, the gelation time can be regulated by controlling the concentration of ethanol, achieving control of GOT from 10 s to 100 s. Although a lower amount of ethanol will speed up the gelation process, an insufficient ethanol concentration will prevent PVA and TA from dissolving properly, leading to an inhomogeneous system.

Figure 2d shows the changes in G′ and G″ of PVA-TA hydrogel at different temperatures. It can be seen from the figure that the G′ and G″ at high temperatures are higher than those at low temperatures, indicating that the phase transition of the hydrogel is significantly accelerated at high temperatures. Taking 10 °C as an example, G′ is always less than G″ before t = 86 s. When the time reaches 86 s, G′ = G″ = 30 (Pa), and the two lines intersect. Subsequently, G′ is higher than G″. Figure 2e shows that the GOT is approximately 2.17 s at 40 °C, which is even shorter than the startup time of the rheometer, while the GOT reaches 87.55 s at 10 °C. This may be due to the accelerated evaporation of ethanol at high temperatures, which promotes the reconstruction of hydrogen bonds between PVA and TA molecules.

**Figure 3:**
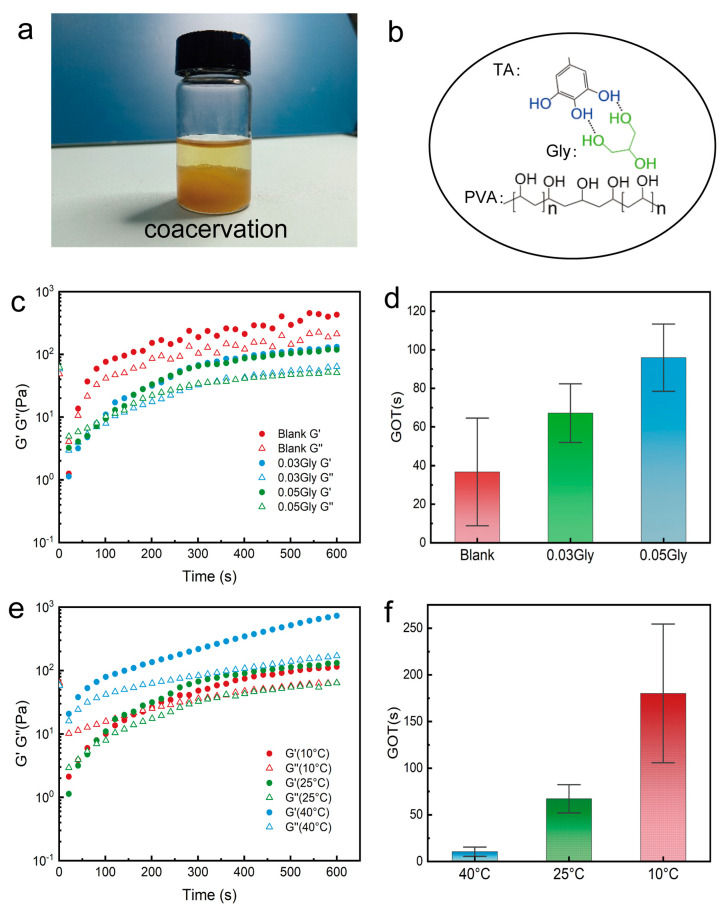
Changes in hydrogel properties under the influence of Gly. (**a**) Liquid-liquid phase separation without ethanol regulation. (**b**) Cross-linking mechanism after adding glycerol. (**c**) Changes in G′ and G″ during gelation with 0.03Gly and 0.05Gly (n = 3~4, *p* < 0.05). (**d**) Changes in GOT with 0.03Gly and 0.05Gly. (**e**) Changes in G′ and G″ during gelation at different temperatures with 0.03Gly (n = 3, *p* < 0.05). (**f**) Temperature dependence of GOT with 0.03Gly.

If ethanol is replaced with other volatile alcohols such as ethylene glycol or glycerol, the dissolution process becomes more challenging. Although the solutes could dissolve after heating at 90 °C for 4 h, liquid-liquid phase separation occurs upon cooling (Figure 3a). This phenomenon is also known as coacervation, which is typically driven by liquid-liquid phase separation due to classical interactions among charged copolymers. Studies have also indicated that hydrogen bonding is one of the causes of coacervation [42,43]. The occurrence of coacervation suggests that such hydrogels may have potential applications in wastewater treatment and other related fields.

Figure 3b shows that introducing glycerol (Gly) into the system competes with PVA for hydrogen bond binding sites on TA, thereby reducing the cross-linking strength. Liu et al. used density functional theory (DFT) to calculate the binding form of TA and glycerol, and found that glycerol can occupy the original binding sites [44].

Figure 3c,d show the effects of added Gly on the storage modulus (G′) and loss modulus (G″) of the hydrogel, as well as the GOT. The hydrogels with 0.03 mol of Gly have lower G′ and G″ values compared to the control group, and the GOT increases by 30 s. As the Gly content increases to 0.05 mol, the GOT further increases to 96 s. This indicates that the addition of Gly slows down the phase transition rate and prolongs the gelation time. Figure 3e,f illustrate the temperature dependence of GOT for hydrogels with 0.03 mol of Gly. Compared to Figure 2d,e, the GOT increases significantly at different temperatures: by 8 s (386.5%) at 40 °C, by 30 s (83%) at 25 °C, and by 99 s (105.7%) at 10 °C. These results suggest that adjusting the temperature can mitigate the increase in GOT caused by the addition of Gly. Figure 3 primarily illustrates the changes in rheological properties and the alteration of GOT after the addition of Gly. The introduction of Gly leads to an increase in GOT, which is not what we want. However, by adjusting the temperature to 25 °C, the GOT can be reduced to around 70 s.

**Figure 4:**
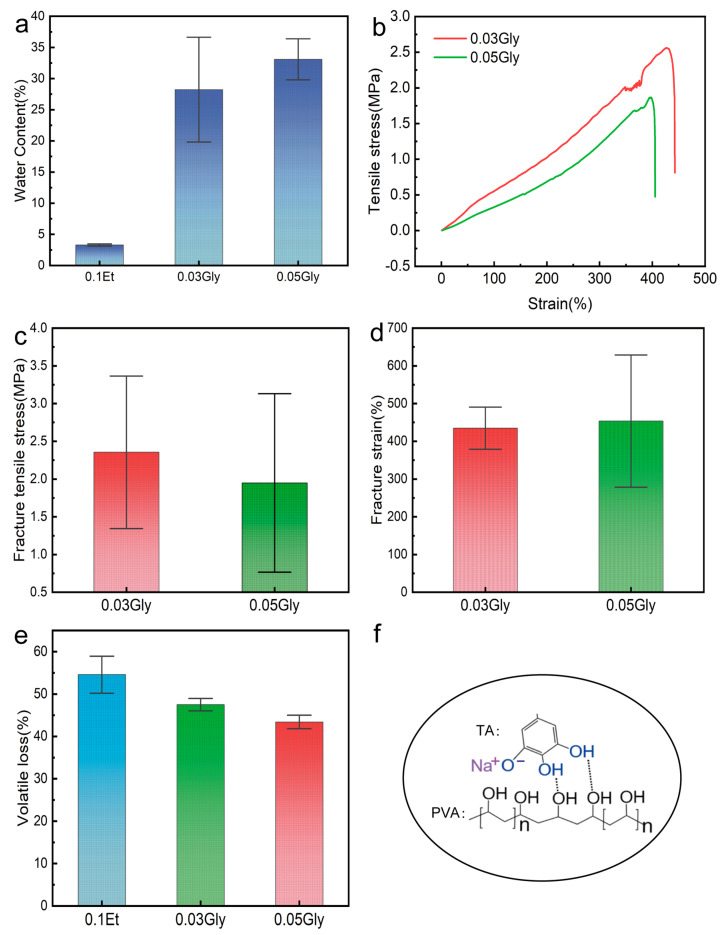
Testing of water retention and mechanical properties of hydrogels under the influence of 0.03 mol Gly and 0.05 mol Gly. (**a**) Water retention test of Gly-added hydrogels (n = 3, *p* < 0.05). (**b**) Classic stress-strain curves after adding Gly. (**c**) Ultimate tensile stress test with different Gly concentrations (n = 5~9, *p* < 0.05). (**d**) Elongation at break test with different Gly concentrations (n = 5~9, *p* < 0.05). (**e**) Volatility of the hydrogel after 5 h with Gly added (n = 3, *p* < 0.05). (**f**) Cross-linking mechanism after adding NaOH.

Adding Gly can significantly enhance the water retention capacity of the hydrogels and keep most of their mechanical strength [45]. Figure 4a illustrates the water retention of the hydrogels after Gly addition. After gelation and resting for 20 h, the water content of the 0.03Gly sample reached 28%, which is 758% higher than that of the control group (3.3%). The 0.05Gly sample had a water content 905% higher than the control group. Figure 4b–d show the mechanical properties of PVA-TA hydrogels with different concentrations of Gly added at 25 °C. As shown in Figure 4b, the hydrogel samples with added Gly exhibit better tensile strain, which is related to the water retention effect of Gly and its regulation of hydrogen bonds within the hydrogel. Due to the water retention effect of Gly, the stretched samples change from a film state to a hydrogel state. During the stretching process, the dynamic breaking and forming of hydrogen bonds can effectively dissipate energy and prevent crack propagation. The presence of Gly provides more hydrogen bond donors and acceptors within the system, increasing energy dissipation during stretching [46]. Additionally, while the addition of Gly significantly enhances the tensile properties of the hydrogel, it reduces the tensile stress.

As shown in Figure 4c, the fracture tensile stress is 2.35 MPa after the addition of 0.03 mol to Gly. However, previous studies have shown that PVA-TA hydrogels possess high tensile strength (>7 MPa) and low strain (<60%) [16]. This is because Gly occupies some of the phenolic hydroxyl groups, disrupting the original cross-linking sites between PVA and TA and reducing the concentration of hydrogen bonds involved in cross-linking. When the Gly content is further increased, the fracture tensile stress of the hydrogel decreases further. Figure 4d shows that the fracture strain of the hydrogel increases with the addition of Gly. In the 0.03Gly sample, the fracture strain increases to 435%. On one hand, the introduction of Gly reduces the volatile loss in the system, leading to an increased cross-sectional area of the stretched samples. On the other hand, the presence of more hydrogen bond donors and acceptors may cause the hydrogel to break the original cross-linking hydrogen bonds and form new ones under stress, thereby repairing minor fractures within the hydrogel. Taking into account both the gelation time and tensile properties, we selected the 0.03Gly sample for further experiments.

Figure 4e illustrates the effect of Gly on ethanol evaporation. It can be observed that the volatile loss of the 0.03Gly and 0.05Gly samples decreased by 7% and 11%, respectively. This result indicates that increasing the amount of Gly can slow down the evaporation rate of ethanol, which in turn implies an increase in the gelation time. Moreover, the evaporation rate decreases further with increasing Gly content. This is because the addition of glycerol reduces the saturated vapor pressure of the solvent, thereby reducing the evaporation rate [45].

**Figure 5:**
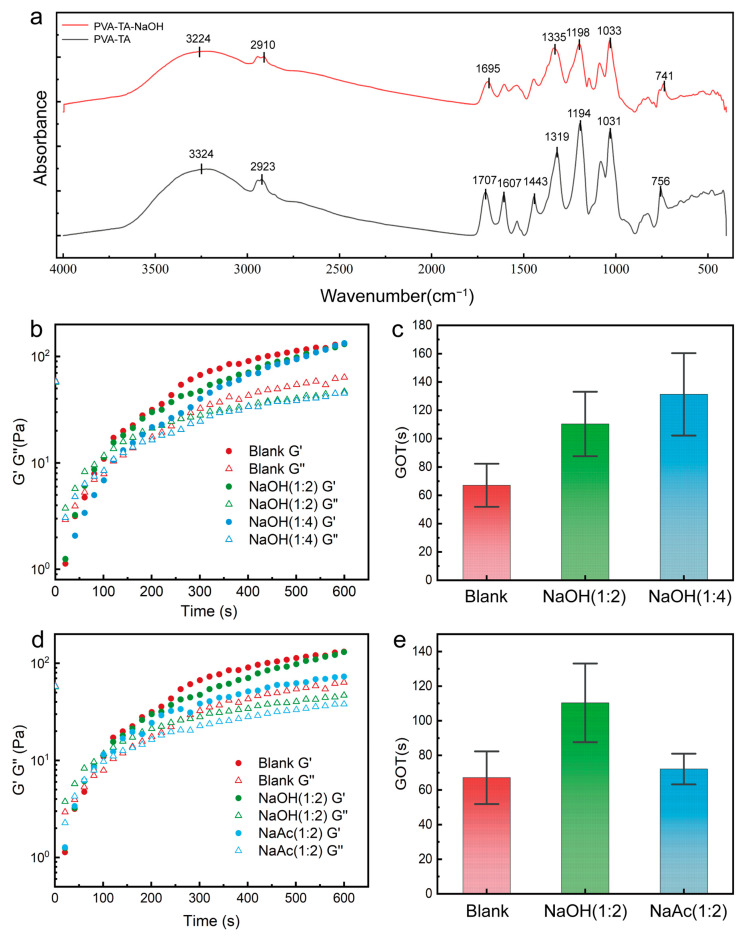
Fourier transform infrared spectroscopy and rheological testing of hydrogels after adding NaOH and NaAc. (**a**) FTIR spectra of hydrogels. (**b**) Changes in G′ and G″ during gelation with added NaOH. (**c**) Changes in GOT with added NaOH (n = 3~6, *p* < 0.05). (**d**) Changes in G′ and G″ during gelation with added NaAc. (**e**) Changes in GOT with added NaAc (n = 3, *p* < 0.05).

Due to the presence of phenolic hydroxyl groups, TA exhibits weak acidity (pKa ≈ 8.5), and the addition of NaOH to the system promotes the ionization of TA, as shown in Figure 4f. The form of the three phenolic hydroxyl groups on the terminal chain of TA was considered for alteration. NaOH was added at molar ratios of TA: NaOH of 1:2 and 1:4, resulting in different extents of ionization of TA. Figure 5a presents the FTIR spectra of PVA-TA hydrogels adjusted with NaOH, excluding the interference of Gly, in the wavenumber range of 400 to 4000 cm^−1^. The characteristic peak of phenolic hydroxyl groups at 3324 cm^−1^ is weakened, indicating that some hydroxyl hydrogen atoms have been ionized. The absorption peaks of the C-O bonds at 1198 cm^−1^ and 1031 cm^−1^ have undergone a slight blue shift, indicating a decrease in hydrogen bond strength. It is worth noting that, after the addition of NaOH, in the frequency range of 1000 to 1500 cm^−1^, the originally sharp and strong absorption peaks have become broad and of medium intensity. This may be due to the electronic effect causing a change in the force constant of the C-O bonds, resulting in a broadening of the absorption frequencies. It may also promote stress transfer, thereby improving the tensile strength and modulus of composite materials [47,48]. The absorption peak at 741 cm^−1^ comes from the C-H bond on the benzene ring, while the absorption peaks of C=O at 1607 cm^−1^ and 1443 cm^−1^ are weakened. The C-O bond absorption peak at 1319 cm^−1^ shifts to 1335 cm^−1^.

Figure 5b,c show the changes in G′ and G″ as well as GOT after the addition of different concentrations of NaOH. Figure 5b illustrates that after adding NaOH at a molar ratio of 1:2, the focal point of G′ and G″ was delayed, indicating a slower gelation rate. Figure 5c shows the GOT of samples with different ratios of NaOH. The GOT of the sample with NaOH(1:2) increased by 43 s compared to the blank, while the sample with NaOH(1:4) increased to 131 s. This may be due to the induced polarity change of ethanol molecules under the influence of charge, which in turn slowed down the evaporation rate. Additionally, the hydroxide ions reacted with hydrogen ions, reducing the number of hydrogen bond donors on the TA molecules and consequently decreasing the number of hydrogen bonds. As shown in Figure 5d,e, when NaOH was replaced with NaAc, the GOT only increased by 5 s. This is because NaAc is weaker in alkalinity compared to NaOH, and the reduction in hydrogen bond donors is almost negligible.

**Figure 6:**
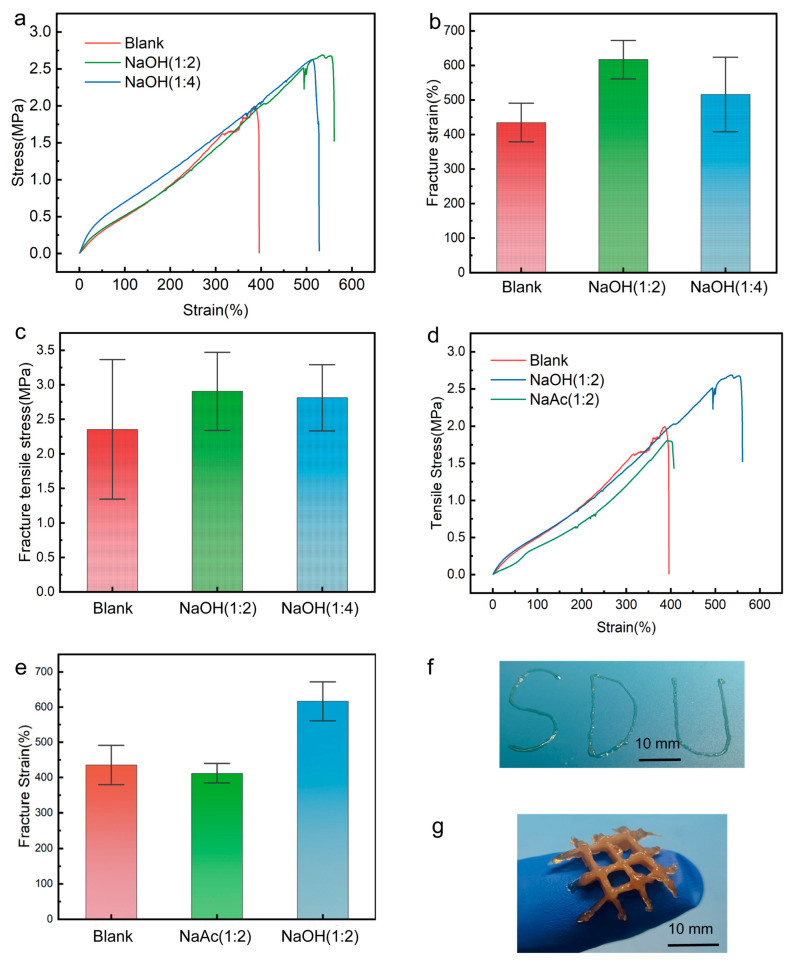
Mechanical property testing of hydrogels and 3D printing patterns. (**a**) Classic stress-strain curves after adding NaOH. (**b**) Elongation at break after adding NaOH (n = 6~9, *p* < 0.05). (**c**) Ultimate tensile stress after adding NaOH (n = 6~9, *p* < 0.05). (**d**) Classic stress-strain curves after adding NaAc. (**e**) Elongation at break after adding NaAc (n = 6~9, *p* < 0.05). (**f**) Extrusion demonstration after 24 h of rest with excess NaAc. (**g**) Printing of 3D hydrogels by layering.

Figure 6a–c show the mechanical property tests of the hydrogel after adding NaOH. Figure 6a represents the typical stress-strain curves during the tensile process, where all samples exhibit good fracture tensile stress (>2 MPa) and fracture strain (>400%). Figure 6b shows a significant improvement in the tensile properties of the gel after adding NaOH, with the fracture strain increasing from 435% to 617%. This may be due to the PVA chains beginning to entangle under the influence of charge, causing the PVA-TA gel to transition from a highly cross-linked gel to a highly entangled hydrogel [49]. As shown in Figure 6c, the increase in tensile fracture stress from 2.35 MPa to 2.90 MPa for the NaOH(1:2) sample suggests that NaOH addition may enhance the cross-linking strength between PVA and TA. Additionally, Figure 6b,c show that when the ratio of NaOH increases from 1:2 to 1:4, both the fracture tensile stress and fracture strain of the hydrogel decrease. This is because, at a lower concentration of NaOH, TA acts as a weak acid with some buffering capacity, maintaining the pH of the solution relatively stable. When the amount of NaOH is doubled, the concentration of H^+^ ions rapidly decreases, leading to a reduction in hydrogen bond donors in the system and a weakened ability for dynamic breaking and forming of hydrogen bonds. As shown in Figure 6d,e, when NaOH is replaced with NaAc, the weak basicity of NaAc has a minimal impact on the H^+^ concentration in the system. Moreover, the acetate ions may lead to a slight decrease in the hydrolysis degree of PVA, resulting in a minor reduction in the hydrogel’s fracture strain and fracture tensile stress (by approximately 5%). Due to the lower stability of TA in alkaline conditions and the prolonged high-temperature heating during the preparation of the hydrogel precursor, TA may undergo thermal decomposition.

In order to compare the effects of NaOH and NaAc on the hydrogel, an excessive amount of NaAc was added to the system to achieve the same effect as NaOH. In the experiment, solutions with molar ratios of n(TA):n(NaAc) = 1:40 and 1:50 were prepared. After resting for 3–4 days (If the amount of NaAc is decreased, then this time will be increased), the solutions spontaneously formed hydrogels. Preliminary experiments indicate that these hydrogels had extrudability and could be used for controlled patterning (as in Figure 6f). This result may be attributed to acetate ions reducing the degree of hydrolysis of PVA, thus enhancing the entanglement between PVA molecules. After resting the pattern for 10 h, the ethanol in the system completely evaporated, and the strength of the hydrogel significantly increased. The patterning process involves self-cross-linking within the hydrogel system and does not require an initiator, making it potentially useful for 3D printing. As shown in Figure 6g, we fabricated simple 3D hydrogels by layer-by-layer printing. However, too much NaAc may cause TA to oxidize and turn black in the air.

## 3. Conclusions

In summary, we have investigated the impact of hydrogen bonds on PVA-TA-based hydrogels and achieved rapid gelation through the use of ethanol. The introduction of glycerol increases the number of hydrogen bond donors and acceptors within the system. Through the dynamic regulation of hydrogen bonds, the tensile properties of the hydrogel were enhanced by approximately 300%, although the gelation time correspondingly increased. By adjusting the temperature, the gelation time could be shortened once again. The cross-linking mechanism between PVA and TA is inferred from the experiments, and it is predicted that the addition of NaOH would reduce the strength of hydrogen bonds. The experimental results show that the optimized hydrogel possesses high fracture tensile stress (>2 MPa) and good fracture strain (>600%). Moreover, the addition of excess NaAc demonstrates potential application value for the hydrogel in the field of 3D printing. This study proposes and verifies the effects of hydrogen bonds in PVA-TA hydrogels on gelation speed, tensile strength, and tensile properties, providing a basis for the development of PVA-TA-based hydrogels in the field of rapid prototyping of high-strength hydrogels and proposing optimization strategies for the tensile properties of these hydrogels.

## 4. Materials and Methods

### 4.1. Materials

Tannic acid (TA, MW~1701.2 g/mol) was purchased from Aladdin Inc. (Shanghai, China). Polyvinyl Alcohol 1799 (PVA, degree of alcoholysis 98~99%) was obtained from Macklin Biochemical Technology Co., Ltd. (Shanghai, China). Anhydrous ethanol, Glycerol and ethylene glycol (EG, purity > 99%) were purchased from Fuyu Fine Chemical Co., Ltd. (Tianjin, China). NaOH and Anhydrous sodium acetate were purchased from Sinopharm Chemical Reagent Co., Ltd. (Shanghai, China).

### 4.2. Preparation of the PVA-TA Hydrogels

Based on previous experience, 1 g of PVA was mixed with an equal mass of TA and used as the solute [16]. The mixtures were dissolved in solvents with the molar ratios of ethanol to deionized water being 0.075:0.5, 0.1:0.5, and 0.125:0.5, and the molar ratio of isopropanol to deionized water being 0.1:0.5. The solutions were heated in a water bath at 85 °C for 2 h to dissolve. The resulting solutions were labeled as 0.075Et, 0.1Et, 0.125Et, and 0.1IPA, respectively.

Referring to David Hardman et al. for the ratio of water to Gly, the prepared 0.1Et sample was separately added with 0.03 mol of Gly and 0.05 mol of Gly [50]. The mixtures were dissolved by heating in a water bath at 85 °C for half an hour. The resulting samples were labeled as 0.03Gly and 0.05Gly, respectively.

TA has multiple ionization constants: pKa_1_ = 5.6, pKa_2_ = 6.9, and pKa_3_ = 8.1. In order to ionize the phenolic hydroxyl group in TA, the prepared 0.03Gly sample was separately added with NaOH to achieve molar ratios of n(TA):n(NaOH) = 1:2 and 1:4. The mixtures were dissolved by heating in a water bath at 85 °C for half an hour. The resulting samples were labeled as NaOH(1:2) and NaOH(1:4), respectively.

The prepared 0.03Gly sample was separately added with NaAc to achieve a molar ratio of n(TA):n(NaAc) = 1:2. The mixture was dissolved by heating in a water bath at 85 °C for half an hour. The resulting sample was labeled as NaAc(1:2).

### 4.3. Characterization of the Gelation Process at the Macroscopic Level

Macroscopic observations of the gelation behavior of the samples were conducted. A volume of 0.2 mL of the 0.1Et sample was pipetted into a petri dish, and its changes were recorded at regular intervals.

### 4.4. Characterization of Gelation Time

The gelation time of the samples was characterized by rheological testing. A rheometer (MCR 302, Anton Paar, Graz, Austria) was used to measure the storage modulus (G′) and loss modulus (G″) of the hydrogel at a strain rate of 1% and a scanning frequency of 1 Hz.

### 4.5. Mechanical Property Testing

The tensile strength and strain rate of the hydrogel were tested using a universal testing machine (ZLC-2D, Jinan XLC Testing Machine, Jinan, China). Specimens were prepared using a dumbbell-shaped mold with dimensions of 4 mm × 1 mm. The samples were filled with the hydrogel solution and then left undisturbed for 20 h. After this period, the samples were taken out for tensile testing. It should be noted that, due to the gelation being induced by solvent evaporation, the thickness of the samples may decrease slightly during the gelation process.

### 4.6. Volatility and Water Retention Testing

The volatility of ethanol and the water retention capacity of the hydrogels were tested through weight loss experiments. For the volatility test, equal amounts of the samples 0.1Et, 0.03Gly, and 0.05Gly were placed in petri dishes and left undisturbed for 4 h, after which the weight loss was recorded [51].

For the water retention test, equal amounts of the gel samples 0.1Et, 0.03Gly, and 0.05Gly were placed in a well-ventilated area and left undisturbed for 20 h. The samples were then subjected to freeze-drying, and the water content was measured [52].

### 4.7. FTIR Spectra Test

The characteristic functional groups in the hydrogels were identified using infrared spectroscopy. The hydrogel samples were first subjected to freeze-drying to remove any residual water or solvent. Subsequently, the infrared spectra were recorded using an infrared spectrometer (Thermo Fisher Scientific Nicolet iS5, WI, America ) in the wavenumber range of 4000–400 cm^−1^ [16]. The scan resolution is 4 cm^−1^ and the number of scans is 32.

### 4.8. Preparation for 3D Printing

1 g of polyvinyl alcohol (PVA) was mixed with an equal mass of triethanolamine (TA) and was used as the solute. The mixture was dissolved in a solvent with a molar ratio of ethanol to deionized water of 0.1:0.5 and a molar ratio of glycerol to deionized water of 0.03:0.5. The molar ratio of TA to sodium acetate (NaAc) was 1:40. The solution was heated in a water bath at 85 °C for 2 h to dissolve it, then left to stand at room temperature for 3–4 days. Hydrogels were extruded using syringes or 3D printers (EFL-BP-6602 printer, Suzhou, China).

Each sample was tested three or more times, and obvious outliers were removed. Data processing was performed using Origin 2022. Use ‘n’ to represent the number of repeated independent experiments.

Table with the Average ± SEM is shown in the Supplementary Materials.

## Data Availability

The original contributions presented in this study are included in the article/Supplementary Materials. Further inquiries can be directed to the corresponding author.

## Supplementary Materials

The following supporting information can be downloaded at: https://www.mdpi.com/article/10.3390/gels11080602/s1, Statistical Data in the Article.

## Nomenclature

PVA	Polyvinyl alcohol	NaOH	Sodium hydroxide
TA	Tannic acid	NaAc	Sodium acetate
Et	Ethanol	G′	Storage modulus
Gly	Glycerol	G”	Loss modulus
IPA	Isopropanol	GOT	Gelation onset time
Ratio Nomenclature In Sample			
0.075Et	n(Et):n(H_2_O) = 0.075:0.5	0.05Gly	n(Gly):n(H_2_O) = 0.05:0.5
0.1Et	n(Et):n(H_2_O) = 0.1:0.5	NaOH(1:2)	n(TA):n(NaOH) = 1:2
0.125Et	n(Et):n(H_2_O) = 0.125:0.5	NaOH(1:4)	n(TA):n(NaOH) = 1:4
0.1IPA	n(IPA):n(H_2_O) = 0.1:0.5	NaAc(1:2)	n(TA):n(NaAc) = 1:2
0.03Gly	n(Gly):n(H_2_O) = 0.03:0.5		
