# Supplementary material for: Hydrogen Bond-Regulated Rapid Prototyping and Performance Optimization of Polyvinyl Alcohol–Tannic Acid Hydrogels
Source: Gels. 2025 Aug 1;11(8):602. doi: 10.3390/gels11080602 (PMC12385280; doi:10.3390/gels11080602)
Supplement: Supplementary file 1 [file gels-11-00602-s001.zip › gels-3731031-supplementary.pdf]

Hydrogen Bond-Regulated Rapid Prototyping and Performance Optimization of Polyvinyl Alcohol - Tannic Acid Hydrogels

Xiangyu Zou and Jun Huang *

Key Laboratory of High Efficiency and Clean Mechanical Manufacture of Ministry of Education, School of Mechanical Engineering, Shandong University, Jinan, Shandong, 250061, China
* Correspondence: Email: jun.huang@email.sdu.edu.cn

Statistical Data in the Article

Figure	Sample	Average	SEM
**Figure 2c**	0.075Et	7.1	2.6
	0.1Et	36.7	27.9
	0.125Et	99.4	9.8
	0.1IPA	96.4	20.8
**Figure 2e**	10℃	87.6	6.4
	25℃	36.7	27.9
	40℃	2.2	0.03
**Figure 3d**	blank	36.7	27.9
	0.03Gly	67.1	15.2
	0.05Gly	95.9	17.5
**Figure 3f**	10℃	180.1	74.3
	25℃	67.2	15.2
	40℃	10.6	4.9
**Figure 4a**	0.1Et	3.3	0.2
	0.03Gly	28.2	8.4
	0.05Gly	33.2	3.3
**Figure 4c**	0.03Gly	2.4	1.0
	0.05Gly	1.9	1.2
**Figure 4d**	0.03Gly	434.8	55.9
	0.05Gly	453.6	175.1
**Figure 4e**	0.1Et	54.6	4.4
	0.03Gly	47.5	1.5
	0.05Gly	43.4	1.6
	blank	67.1	15.2

**Figure 5c and e**	1:2NaOH	110.3	22.7
	1:4NaOH	131.2	29.2
	1:2NaAc	72.1	8.8
**Figure 6c**	blank	2.4	1.0
	1:2NaOH	2.9	0.6
	1:4NaOH	2.8	0.5
**Figure 6b and e**	blank	434.8	55.9
	1:2NaOH	616.7	55.7
	1:4NaOH	515.9	107.8
	1:2NaAc	411.6	27.4
